# Quantifying the effects of delays on telerobotic surgical operability via brain activity measurements

**DOI:** 10.1007/s11548-025-03487-x

**Published:** 2025-08-04

**Authors:** Junnosuke Ichihara, Satoshi Miura

**Affiliations:** Department of Mechanical Engineering, School of Engineering, Institute of Science Tokyo, Tokyo, Japan

**Keywords:** Telerobotic surgery, Communication delay, Brain activity analysis, Intraparietal sulcus, Operability, Move-and-wait strategy

## Abstract

**Purpose:**

Telesurgery, increasingly valued for enabling remote procedures post-COVID, can be critically affected by communication delays—typically negligible in conventional robot-assisted surgery due to surgeon–patient co-location. While previous studies have assessed the impact of delays on surgical performance, their effects on the operator’s cognitive state remain unclear. Therefore, this study assessed delay-induced changes in telesurgery operability based on intraparietal sulcus (IPS) activity.

**Methods:**

A virtual-reality-based surgical assistance simulator was developed using the Unity game engine to replicate the da Vinci surgical robot and colorectal suturing environment. The simulator randomly introduced seven delay conditions to assess their impact on IPS activity during suturing. Eight right-handed participants, all of whom were non-medical students with no prior surgical experience, performed suturing while their IPS activity was measured using functional near-infrared spectroscopy. The left- and right-sided IPS activities were measured separately, and the task completion time and suturing error rate were also recorded for comparison.

**Results:**

Significance was assessed using the nonparametric Jonckheere–Terpstra test. Left- and right-sided IPS activities decreased significantly for 150–300 and 0–300 ms delays, respectively. The task completion time increased significantly for 0–300 ms delays, while the suturing error rate increased significantly for 0–100 ms delays.

**Conclusion:**

These findings confirm that IPS activity can be used to quantify delay-induced operability changes. For delays beyond 150 ms, significant IPS changes indicated that operators perceived degraded control. However, for delays of or shorter than 150 ms, the operators’ precision unconsciously declined, indicating that greater caution is required in surgical tasks.

## Introduction

Telerobotic surgery is a leading example of technological advancement in medicine and medicine–engineering collaboration. A notable milestone was the Lindbergh operation performed by Marescaux et al. in 2001 [1], in which surgeons in North America and a patient in Europe were connected via telecommunications. Although long-distance telesurgery remains technically challenging and is not part of routine clinical practice, telerobotic systems have since been widely applied in local settings, including in orthopedic and gastric cancer surgery [2, 3]. Following the COVID-19 pandemic in the early 2020s, the need for performing surgeries without direct physical contact has increased, further driving the demand for remote surgical procedures [4].

In telerobotic surgery, robots employ teleoperation technology known as the leader–follower system, where the surgeon operates a controller while viewing images from an endoscope, allowing for the precise manipulation of a remotely located robotic arm and enabling movements more complex than those of the human hand by suppressing tremor and expanding movements through control [2]. Moreover, even if the surgical site is in a disaster-affected or remote area, telerobotic surgery enables high-precision procedures without requiring the surgeon’s physical presence [5]. Where surgeries span continents, telerobotic surgery eliminates the need for travel by either the physician or the patient, potentially alleviating the current global shortage of medical professionals.

However, one of the primary challenges of telesurgery is communication delay, which introduces temporal lag (delay) that disrupts synchronization among the surgeon’s hand movements, robotic arm’s response, and visual feedback received by the surgeon, ultimately impairing operability. While such delays are generally negligible in conventional robot-assisted minimally invasive surgery (RAMIS) due to the co-location of the surgeon and patient, the delay become critical in telesurgery, where long-distance communication is required. This issue is not unique to telesurgery; similar concerns have been raised in planetary robotics, where low-latency telepresence has been proposed to preserve human cognitive performance during remote operations [6]. In surgical robotics, latency has likewise been recognized as a major technical barrier to safe and effective long-distance procedures [7]. Although advancements in rapid communication technologies, such as 5G, have reduced delay, the quantitative assessment and establishment of precise thresholds remain crucial for evaluating the magnitude of delay’s negative impacts on surgical operability. In medicine, because even minor errors or malfunctions can have life-threatening consequences, the evaluation of the leader–follower system’s performance is essential using accurate and reliable metrics.

In telesurgery, the impact of the delay on operability remains unclear. Some studies have evaluated this effect based on objective performance metrics, such as the surgical completion time and trajectory length of forceps tips [8, 9]. Others have assessed operability based on subjective measures, including the National Aeronautics and Space Administration’s task load index (NASA-TLX) and similar survey-based evaluations [10, 11]. However, to the best of our knowledge, few studies have investigated this issue from the perspective of the operator’s cognitive experience. Because surgical operability is inherently linked to the surgeon’s sensory and motor experiences, a comprehensive evaluation requires objective and quantitative metrics that can be used to assess how delays affect the surgeon’s perception and control accuracy.

One promising approach is the use of brain activity measurements to evaluate the cognitive experience of surgeons during telesurgery. Brain activity measurement has been explored as a means to assess non-technical skills and cognitive workload in RAMIS. Nagyné Elek et al. conducted a systematic review [12]　and highlighted that despite its clinical importance, the evaluation of mental workload and soft skills in RAMIS remains underexplored, with most existing methods relying on subjective assessments. Meanwhile, Modi et al. systematically reviewed neuroimaging studies involving surgeons and demonstrated that brain activity patterns reflect skill acquisition, cognitive demand, and decision-making processes [13].

In particular, the intraparietal sulcus (IPS), in the parietal lobe, plays crucial roles in integrating spatial information for controlling arm and eye movements and as an interface for regulating the motor system [14]. Previous research by Miura et al. investigated IPS activity during simulated surgery and revealed that IPS activation patterns could be used to optimize configurations for robotic arm positioning and the spatial arrangement of monitors and hand controllers to enhance operability [15, 16]. Because the IPS integrates visual and proprioceptive information, its activity could reflect the coherence between a surgeon’s visual perception of hand movement and their proprioceptive awareness. If a discrepancy arises because of delays in visual feedback from the robotic system, IPS activity may indicate the cognitive burden imposed by such inconsistencies. However, despite its potential, to the best of our knowledge, IPS activity has not yet been utilized to evaluate the effect of the delay on operability in telesurgical systems, and the incorporation of this approach could provide valuable insights into the cognitive limitations of telesurgery and contribute to the development of more intuitive and delay-resilient robotic systems.

**Fig. 1:**
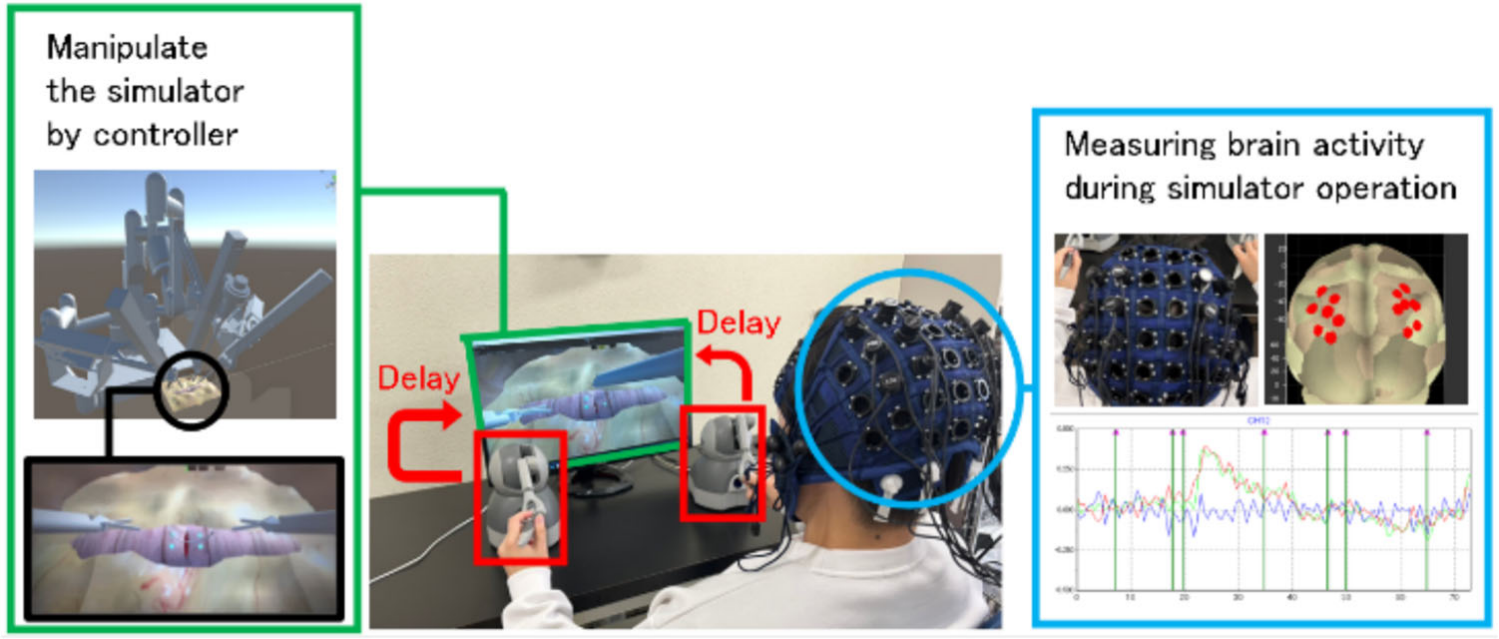
Research framework. The operator manipulates a VR surgical simulator via a controller, and the artificial delay is inserted between the controller input and the monitor output. Simultaneously, the operator's brain activity in the IPS is measured. The IPS responses are then compared across different delay conditions to assess the cognitive impact of communication latency

Therefore, this study quantitatively and objectively evaluated the impact of the delay on telesurgical operability by analyzing IPS brain activity. We developed a virtual-reality (VR)-based surgical simulator and conducted experiments, where participants performed surgical tasks while their IPS brain activity was recorded. The effect of the delay on telesurgical operability was then assessed by comparing brain activation levels, task completion times, and suturing error rates for different delays. The research framework is shown in Fig. 1.

## Method

### Setup

#### Brain activity measurements

**Fig. 2:**
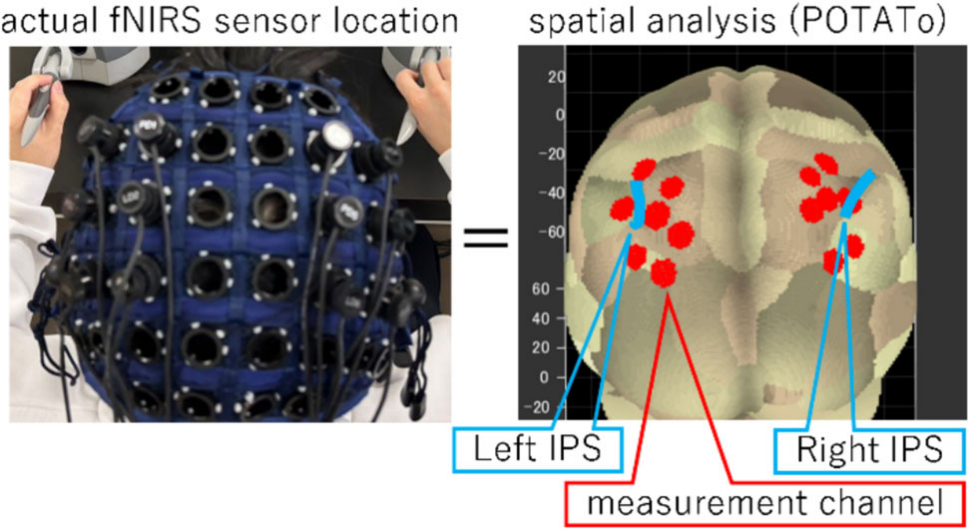
Positions of the IPS and measurement channels

The activity of the IPS (a sulcal structure between the superior and inferior parietal lobules in the parietal lobe, as shown in Fig. 2) was used as a key evaluation measure. According to the automatic anatomical labeling (AAL) system, the IPS is approximately in areas 63 to 66, while Brodmann’s map places it between areas 5 and 7 [17]. The IPS is a part of the parietal association cortex, posterior to the primary somatosensory cortex in the central sulcus and anterior to the occipital lobe, which contains the visual cortex. As a multimodal processing region, the IPS integrates sensory and visual information, particularly by relying on visual cues for controlling hand movements. In both hemispheres, the IPS is bilateral, with the left and right hemispheres predominantly controlling the right and left hands, respectively.

To measure IPS activity, we used functional near-infrared spectroscopy (fNIRS; OEG-17APD system, Spectratech Inc., Tokyo, Japan), a noninvasive optical imaging technique using near-infrared light for measuring changes in regional cerebral blood flow. The fNIRS system operates by emitting near-infrared light into the scalp and detecting the amount of light absorbed by the hemoglobin in the blood. As oxygenated hemoglobin levels fluctuate in response to neural activation, these light absorption changes allow for the indirect measurement of the brain activity. The fNIRS system comprises light emitters and detectors arranged in a grid pattern, with each emitter–detector pair forming a measurement channel. These channels are also shown in Fig. 2. The fNIRS system specifically quantifies changes in oxygenated and deoxygenated hemoglobin levels in the brain region directly beneath the channels. Compared with other neuroimaging methods, fNIRS is relatively simple and cost effective while possessing minimal physical constraints, as it only requires attachment to the head without interfering with limb movements.

For brain activity measurements using fNIRS, the participants first wore a dedicated cap designed to securely attach the fNIRS sensors. The placement of the fNIRS probes was determined using a 3D digitizer (PATRIOT, Polhemus, Colchester, VT) and the PoTATo toolbox in MATLAB (MathWorks, Natick, USA), allowing for precise spatial mapping between the measurement positions and of the participants’ brain regions. The spatial coordinates were further analyzed using the 10–20 international system to estimate the correspondence between the sensor positions and underlying cortical structures.

We specifically targeted the IPS for measurements. The fNIRS cap was adjusted to ensure the optimal alignment with the right- and left-sided IPS regions. However, if the measurement positions and IPS did not precisely overlap because of individual anatomical variations, adjustments were made to capture the activity from the adjacent regions. Specifically, the measurement regions were shifted to encompass both the superior and inferior parietal lobules, which are functionally associated with the IPS and contribute to visuomotor control.

#### VR surgical simulator

**Fig. 3:**
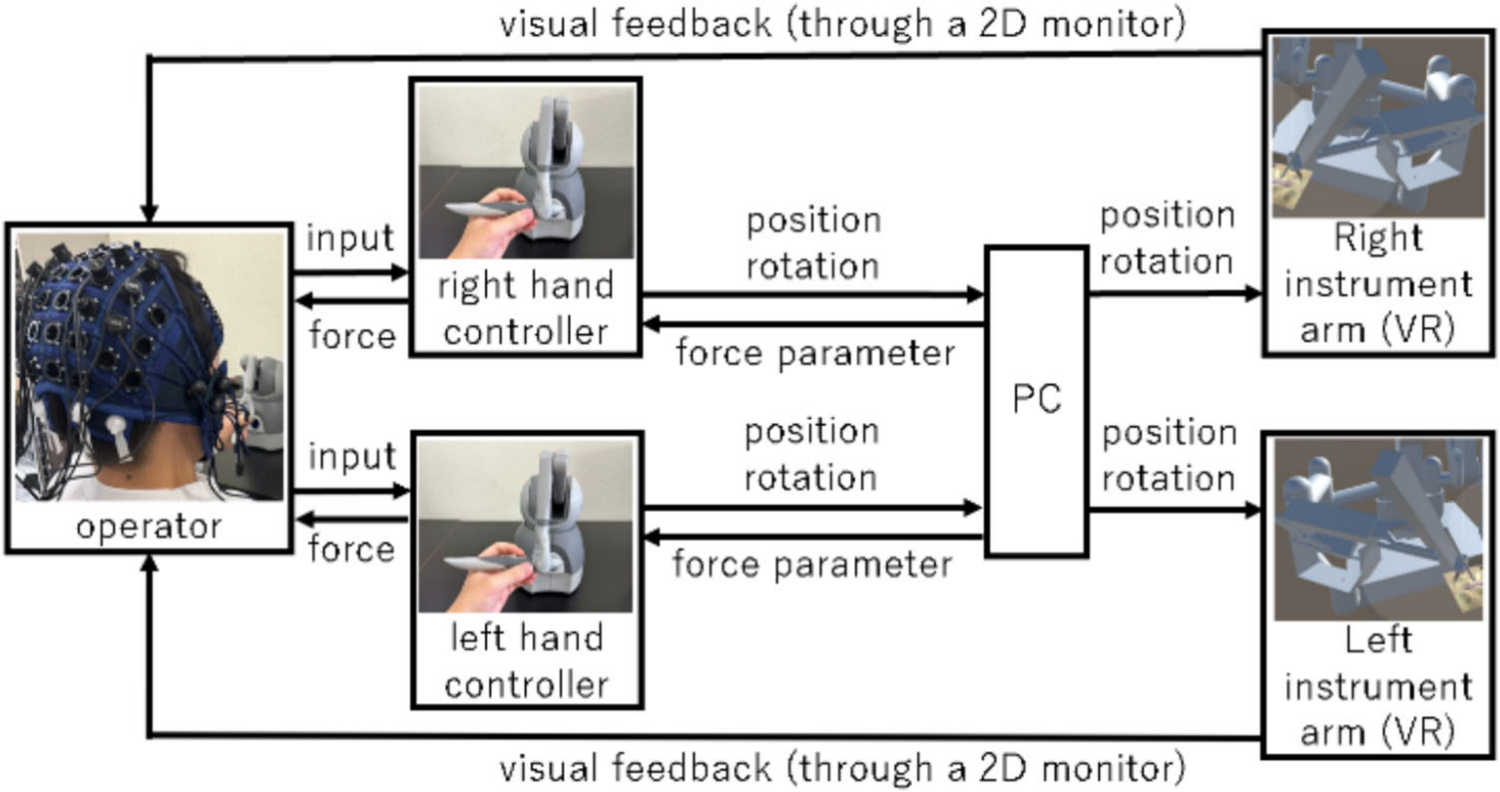
Block diagram of the VR simulator

We also used the Unity game engine (Unity Technologies, San Francisco, USA) to develop a VR-based surgical simulator to investigate the effects of the delay on surgical operability. The simulator enables participants to perform surgical tasks while recording their IPS activities. The block diagram of the simulator is shown in Fig. 3. The operator controls the simulator using two hand controllers simultaneously. We employed one touch device (3D Systems, Rock Hill, USA) for each hand, allowing for six-degree-of-freedom input for the stylus position and orientation while also providing three-dimensional force feedback. When data were output from the personal computer (PC) to the monitor, simulated delay was implemented by introducing a fixed waiting time. The operator received visual feedback, including on the effects of their manipulations, through a 2D monitor. In VR, the robotic arm’s movements were updated based on the operator’s input. Additionally, force feedback was applied based on the motion of the input device and the interaction with virtual objects in the simulator. While haptic feedback is not a standard feature in current clinical robotic systems, force feedback is implemented to represent geometric constraints of the virtual robotic arms, such as touch with an organ. Without force feedback, the trajectory of movement depends on only the input controller, ignoring influence by touch and interference of surgical robot with organs in the working space. Therefore, force feedback was implemented to assist in conveying the movement limitations of the robotic arms within the VR simulator.

**Fig. 4:**
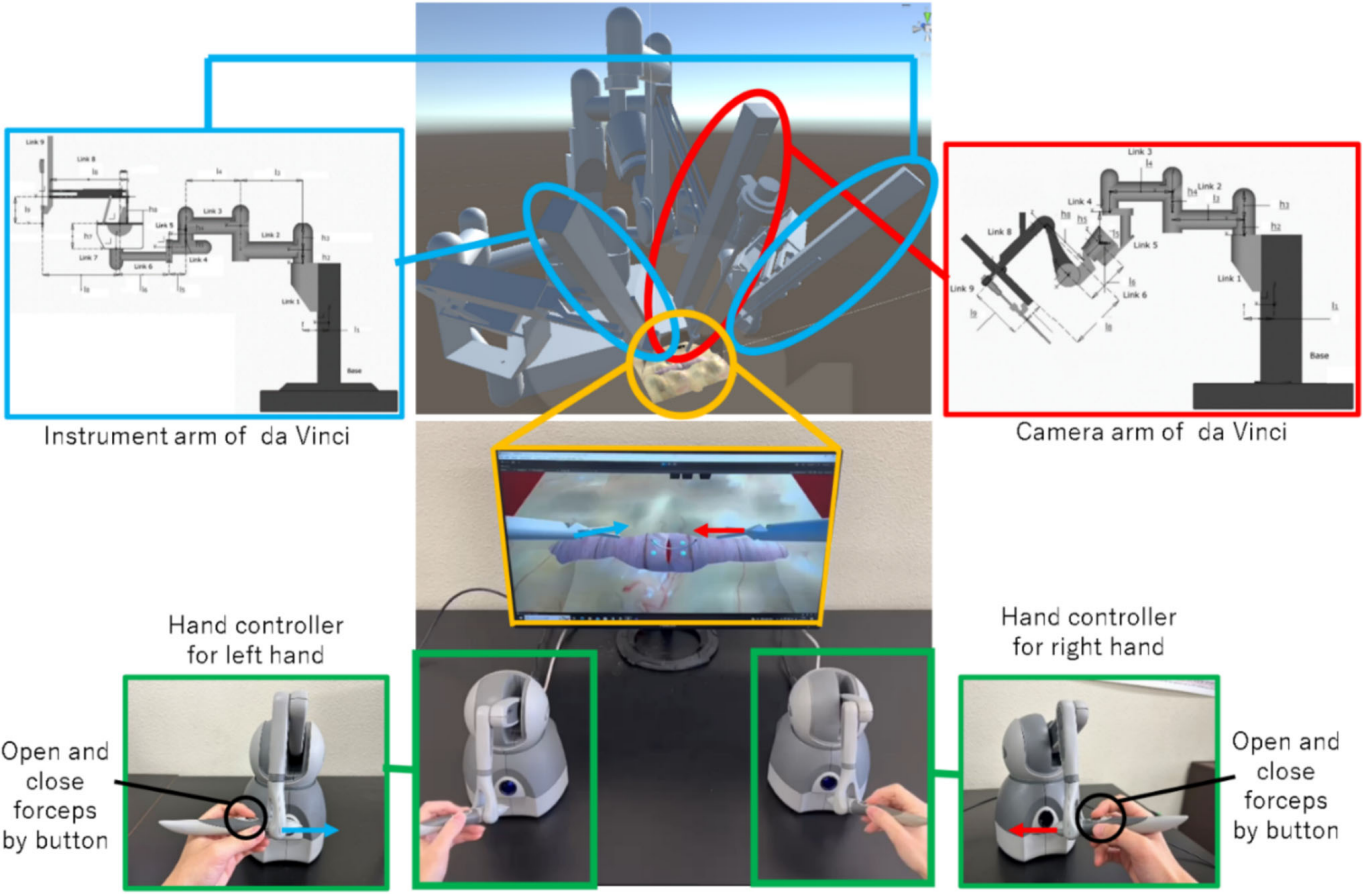
The simulator overview and the relationship between the touch controller and forceps position. The simulator includes two instrument arms and one camera arm, which dimensions were previously measured by Loi et al. [18]

Figure 4 shows the entire VR-simulated surgical environment and the correspondence between the hand controllers’ and robotic arms’ movements in the simulator, where the robotic arms, including two instrument arms and one camera arm each and the essential associated tools, were modeled based on the dimensions previously measured by Loi et al. [18] to replicate the da Vinci surgical robot. The positions and orientations of the instrument arms’ end effectors were associated with the positions and orientations of the styli on the touch hand controllers, respectively.

We simulated a laparoscopic surgical environment as a representative case of RAMIS and replicated the suturing environment for colorectal resection surgery. The da Vinci surgical robot has been increasingly utilized in gastrointestinal surgery, particularly in procedures requiring intracorporeal suturing within a confined surgical space [19]. Among the various tasks involved in colorectal resection, such as suturing, tissue excision, and tissue manipulation, this study focused specifically on suturing because inadequate suturing can lead to complications, such as anastomotic leakage and bowel obstruction, potentially necessitating reoperation [20]. Furthermore, suturing requires fine bimanual coordination and is sensitive to delay-induced disruptions in timing and precision, making it a suitable task for evaluating the cognitive and operational impacts of communication delays. The reaction forces, both when the robot contacted the colon and when a needle was inserted into the colon during suturing, were calculated based on literature values and provided as three-dimensional force feedback to the 3D touch device.

**Fig. 5:**
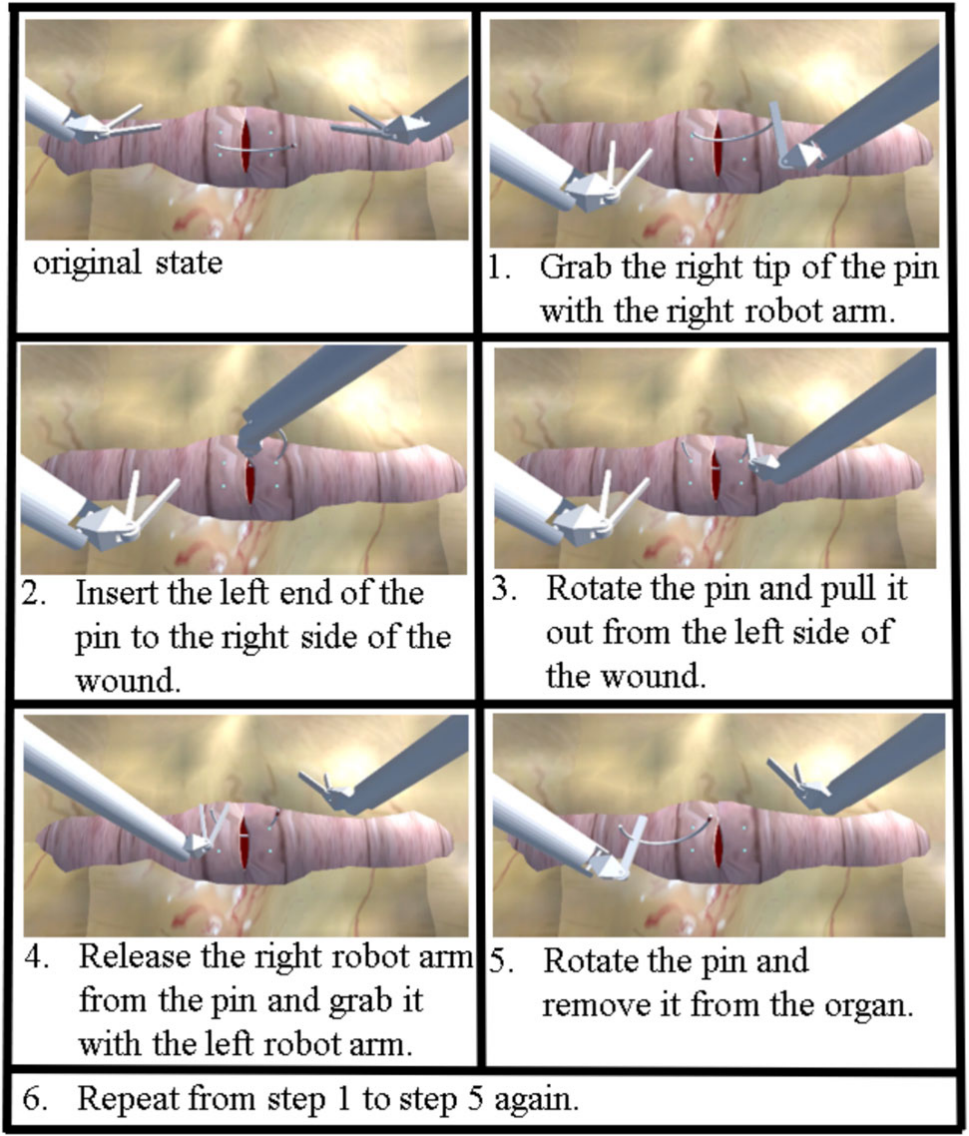
Task procedure

Figure 5 shows the task procedure, involving two suturing operations. The target positions for needle insertion and exit were marked with visual indicators. Before starting the task, the operator set the 3D touch device at its initial position and kept it stationary during the preparation phase. During the task, the completion time and suturing accuracy, defined as the total positional deviation between the actual suturing and target points marked at four insertion and exit locations, were measured. The operator was instructed to complete the suturing as quickly and accurately as possible.

### Procedure

**Fig. 6:**
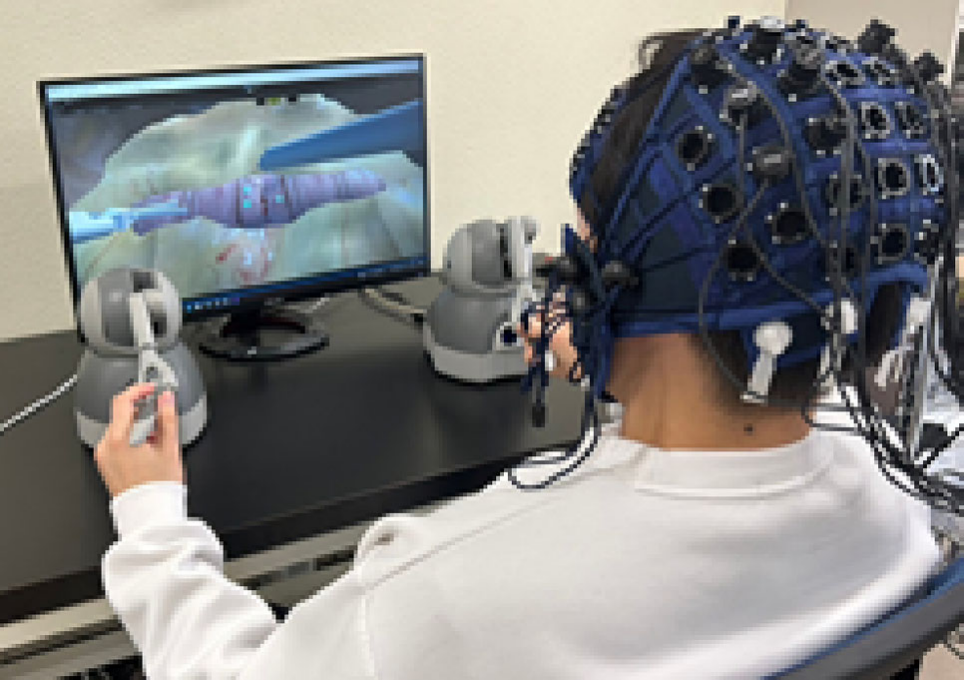
Experimental setting

The experimental setting is shown in Fig. 6. We recruited eight right-handed participants (seven men and one woman) between the ages of 22 and 25, all of whom were non-medical students with no prior surgical experience. Before the main experiment, the participants practiced using the VR simulator and suturing tasks once or twice without delay to familiarize themselves with the system while minimizing learning effects. The participants then performed the VR simulation task while wearing an fNIRS measurement device on their heads. During the experiment, we randomly introduced seven delays (0, 50, 100, 150, 200, 250, and 300 ms) between the controller input and output video to prevent order effects. Each participant repeated the task five times per delay condition, and left- and right-sided IPS activities were separately recorded from task preparation to completion. Additionally, task performance metrics, such as the task completion time and suturing error rate, were measured. To mitigate fatigue effects, participants took a 5-min break after every five trials. After each break, they performed one additional practice round without delay before resuming the main task.

### Analysis

The brain activity data were analyzed using generalized linear modeling (GLM), which has been widely adopted in fNIRS research for evaluating cognitive loads. Suzuki et al. demonstrated that beta values derived from the GLM of block-designed fNIRS data reliably indicate cognitive-processing demand [21]. GLM-based fNIRS analysis has also been utilized in several other studies [22, 23], further supporting its applicability in assessing cognitive workloads during motor tasks.

**Fig. 7:**
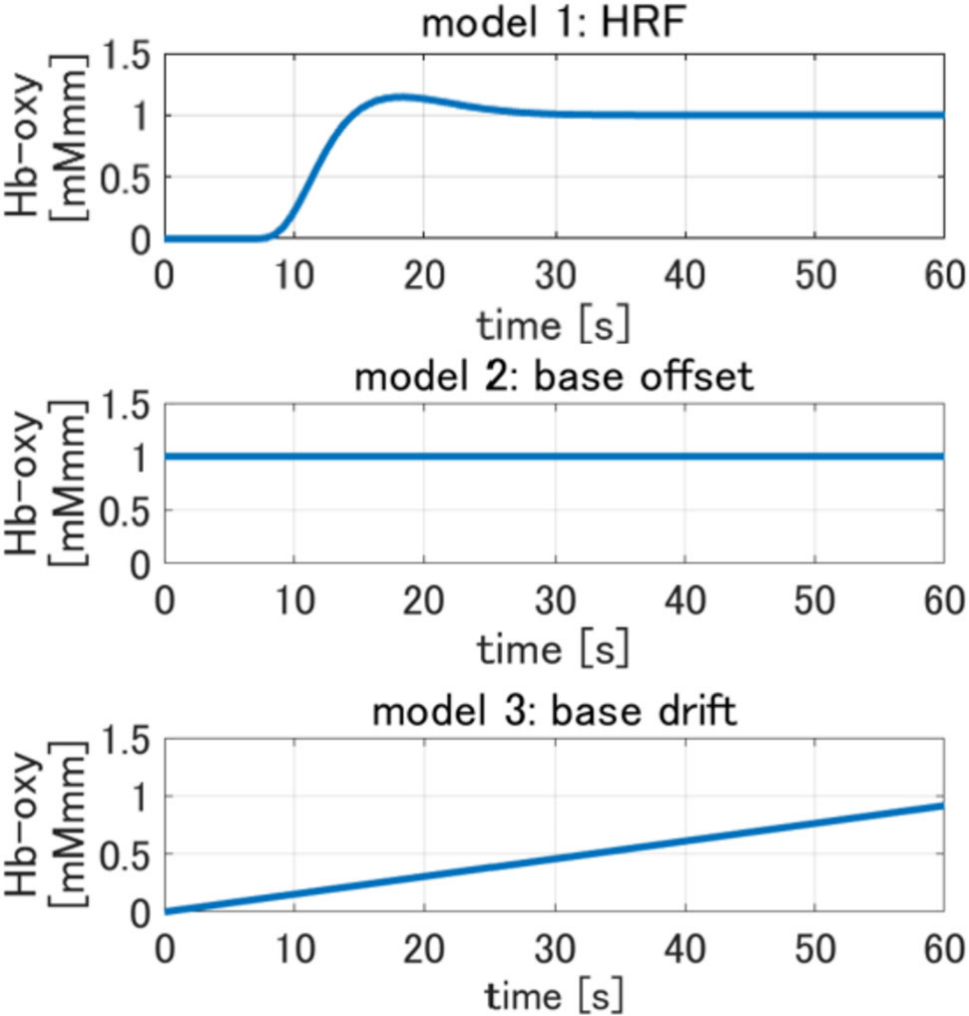
Models used in GLM analysis

Three GLM models were constructed, as shown in Fig. 7. Model 1 involved the convolution of the hemodynamic response function (HRF)—which models the temporal dynamics of the stimulated brain activation, allowing for the extraction of task-related neural responses—with the oxygenated hemoglobin level changes recorded during both the task preparation and execution phases. The data were analyzed using the spm12 toolbox in MATLAB. To account for potential baseline shifts in the fNIRS signal, Models 2 and 3 incorporated constant baseline offset and linearly increasing baseline drift functions, respectively. These additional models helped to control for physiological noise and other slow fluctuations unrelated to task-induced brain activity. The mathematical formula for the GLM analysis is as follows:

1 $$ \begin{array}{*{20}c} {y\left( t \right) = \beta_{1} x_{1} \left( t \right) + \beta_{2} x_{2} \left( t \right) + \beta_{3} x_{3} \left( t \right) + e\left( t \right)} \\ \end{array} $$

Because of the GLM framework, the fNIRS signal ($y(t)$) was modeled as a linear combination of the explanatory variables (${x}_{1}\left(t\right), {x}_{2}\left(t\right)$, and ${x}_{3}\left(t\right)$), corresponding to Models 1, 2, and 3, respectively. Coefficients ${\beta }_{1}, { \beta }_{2}$, and ${\beta }_{3}$ were estimated to minimize the squared error (*e*(*t*)) and optimize the models’ fit to the measured data.

Among the coefficients, ${\beta }_{1}$ is a key brain activity index, indicating the contribution of HRF (Model 1) to the overall response. A higher ${\beta }_{1}$ value indicates stronger task-related neural activation, while ${\beta }_{2}$ and ${\beta }_{3}$ primarily account for baseline fluctuations and drift, respectively. To determine the IPS-specific brain activity, ${\beta }_{1}$ values were assigned based on the overlap degree between the fNIRS measurement regions and the IPS’s anatomical location. If a measurement site directly coincided with the IPS, the corresponding ${\beta }_{1}$ value was used. Where a measurement covered both the superior and inferior parietal lobules, the IPS-associated ${\beta }_{1}$ value was computed as the average ${\beta }_{1}$ value of both lobules, accounting for potential signal dispersion. This approach allowed for a quantitative evaluation of IPS activity across different experimental conditions, enabling an objective assessment of task-related cognitive processing at various delays.

## Results

**Fig. 8:**
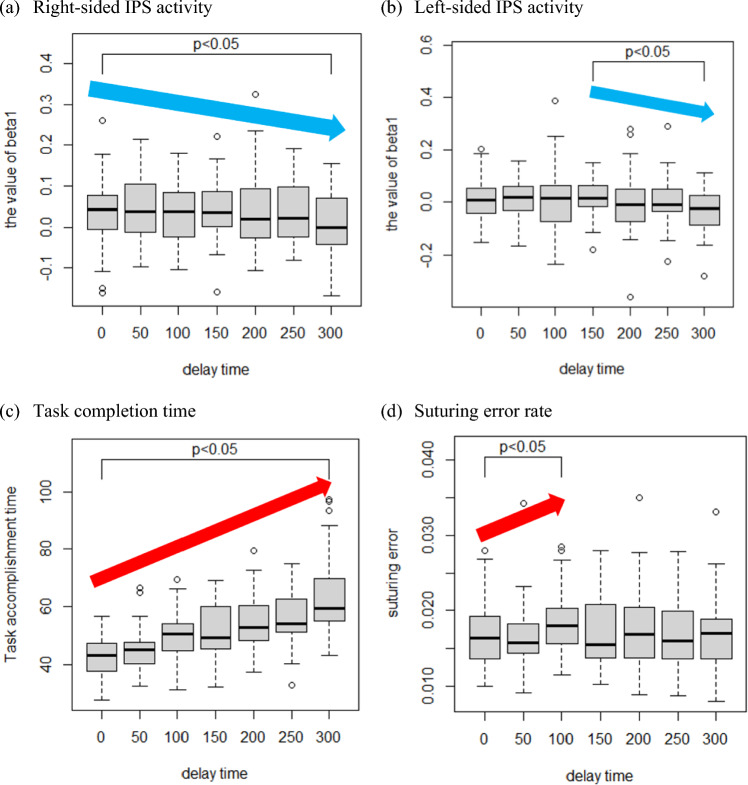
Experimental results under different delay conditions. **a** Right-sided IPS activity significantly decreased across all delay conditions. **b** Left-sided IPS activity significantly decreased across for delays over 150 ms. **c** Task completion time significantly increased across all delay conditions. **d** Suturing error rate significantly increased under 0–100 ms delay conditions

The experimental results are shown in Fig. 8. Figure 8a–d presents boxplots illustrating the distributions of each metric across different delay conditions: (a) right-sided IPS activity, (b) left-sided IPS activity, (c) suturing error rate, and (d) task completion time. For each subplot, arrows are used to indicate delay intervals where statistically significant monotonic trends were observed, clearly visualizing the direction and extent of these changes. During data processing, one participant’s dataset was excluded because of excessive noise caused by head movements and verbalization during the experiment. Therefore, only data from seven participants were analyzed. Statistical significance was evaluated using the Jonckheere–Terpstra test (*p* < 0.05)—a nonparametric method for detecting monotonic trends across multiple conditions—to determine whether the IPS activity, suturing error rate, and task completion time exhibited significant trends with lengthening delay. The results showed that the right-sided IPS activity significantly monotonically decreased across the full delay range (0–300 ms, *p* = 0.010). The Jonckheere–Terpstra statistic (JT statistic), which summarizes the degree to which values follow a consistent increasing or decreasing order across conditions, was 11,354 in the 0–300 ms. In contrast, the left-sided IPS activity significantly monotonically decreased only in the 150–300 ms range (*p* = 0.044, JT statistic = 3214). For surgical performance, the suturing error rate significantly monotonically increased for delays of 0–100 ms (*p* = 0.021, JT statistic = 2787), while the task completion time significantly monotonically increased across 0–300 ms (*p* < 0.001, JT statistic = 22,693). These findings confirm that the delay length systematically influenced both the brain activity and surgical performance metrics. This result shows almost the same trend as the result of Nankaku’s research [8], where delays of 100 ms or less were found to be acceptable based on surgical performance, with degradation becoming more evident beyond that threshold.

## Discussion

The delays at which significant changes (*p* < 0.05) occurred varied between the left- and right-sided IPSs, likely because of differences in hand dominance and brain lateralization. Furthermore, the results indicate that 150 ms is a critical delay threshold, beyond which operators recognize a decline in right-hand operability and adjust their behavior accordingly.

From brain lateralization, right- and left-sided IPSs are associated with the control of the nondominant left and dominant right hands. Because the nondominant hand is usually more difficult to control, it is more susceptible to short delays, which likely explains the early decline in IPS activity. In contrast, the dominant hand is inherently easier to operate, meaning that minor discrepancies between motor input and visual feedback do not immediately degrade surgical operability.

Moreover, for extended delays of 200 and 300 ms, many participants adopted the move-and-wait strategy, commonly observed during long delays in remote operation systems, where the operator slightly moves their hand, waits for visual confirmation, and then proceeds with the next movement [24]. This strategy is not unique to surgical robotics and has been reported in other fields [25, 26].

Considering the impact of the move-and-wait strategy on surgical operability, the relationship between the recognition of IPS-activity-based operability decline and surgical performance can be explained. For short delays (0–100 ms), the right-sided IPS activity remained constant, indicating that operators did not perceive severe right-hand operability degradation. Therefore, although the move-and-wait strategy was not adopted, the suturing error rate increased monotonically with lengthening delay. However, for delays of 150 ms and beyond, the left-sided IPS activity declined, suggesting that right-hand operability degradation became perceptible. Consequently, operators compensated by adopting the move-and-wait strategy, stabilizing the suturing accuracy despite lengthening the delay. Because right-hand movements were closely tied to suturing accuracy, the temporal transitions in the strategy adoption could be analyzed.

However, left-hand operability—indicated by the right-sided IPS activity—continuously declined from the no-delay condition onward, indicating that the move-and-wait strategy was employed, even for short delays. Naturally, because this strategy involves intentional pauses, the task completion time lengthened. Because the left hand adopted the move-and-wait strategy from the outset and the right hand began adopting it for delays beyond 150 ms, the observed monotonically increasing task completion time with lengthening delay is a logical outcome.

It should be noted that this experiment was conducted exclusively with non-medical students, and did not include medical experts. However, as discussed earlier, factors such as hemispheric brain asymmetry, handedness, and the use of the move-and-wait strategy are common human characteristics, and thus the observed trends are likely to be similar even among medical professionals. Nevertheless, the specific delay thresholds at which significant changes occur may differ between non-medical participants and expert surgeons. In future work, we will validate the similarity and difference between novice and experts.

## Conclusion

This study confirms that IPS activity can objectively reflect delay-induced changes in telesurgical operability. We measured the participant’s brain activity using fNIRS, while the participants manipulated a virtual surgical simulator in a variety of the delays. Delays of 150 ms or less increased error rates without changes in left IPS activity, indicating unconscious right dominant hand degradation. For delays longer than 150 ms, left IPS activity significantly decreases without changes in error rates, indicating that operators adapted through move-and-wait strategies to preserve accuracy. These findings suggest that for delays up to 150 ms, surgeons may experience reduced precision without conscious awareness, highlighting the need for caution even under moderate delay conditions.
